# Nomogram-based prediction of hemorrhagic transformation risk integrating platelet-to-white blood cell ratio in patients with acute ischemic stroke after intravenous thrombolysis

**DOI:** 10.3389/fneur.2026.1782110

**Published:** 2026-06-17

**Authors:** Qi Sun, Jiahao Chen

**Affiliations:** Department of Neurology, Affiliated Jinhua Hospital, Zhejiang University School of Medicine, Jinhua, China

**Keywords:** acute ischemic stroke, hemorrhagic transformation, intravenous thrombolysis, nomogram, platelet to white blood cell ratio

## Abstract

**Background:**

Hemorrhagic transformation (HT) is a significant complication in acute ischemic stroke (AIS) patients after intravenous thrombolysis (IVT), frequently leading to unfavorable clinical prognoses. However, the association between the platelet-to-white blood cell ratio (PWR) and the risk of HT, along with its severity subtypes, remains largely unexplored. This study investigates the independent association between low PWR and the increased incidence and severity of HT following IVT.

**Method:**

This study retrospectively included AIS patients who received IVT treatment from our hospital’s stroke database. HT was identified through cranial imaging (CT/MRI) conducted within 24 h post-IVT and categorized into four subtypes based on the European Cooperative Acute Stroke Study (ECASS) criteria. The relationship between PWR and HT was examined using multivariate logistic regression. A restricted cubic spline (RCS) analysis was applied to detect the nonlinear relationship of PWR and HT. Based on variables identified via multivariate logistic regression, a nomogram-based prediction model was developed, and its performance was assessed using receiver operating characteristic (ROC) curves, calibration plots, and decision curve analysis (DCA).

**Results:**

A total of 790 AIS participants were included in this study. Patients who developed HT exhibited significantly lower PWR levels compared to those without HT. The PWR levels were divided into four quartiles: Q1 (<22.0), Q2 (22.0–28.3), Q3 (28.3–36.2), and Q4 (>36.2), respectively. Multivariable logistic regression demonstrated that patients in the highest PWR quartile (Q4) had a 67% reduced risk of HT (odds ratio [OR]: 0.33; 95% confidence interval [CI]: 0.18–0.61). By integrating potential risk factors, the nomogram achieved an AUC of 0.767 (95% CI: 0.716–0.818).

**Conclusion:**

Lower PWR independently correlates with heightened risk and severity of HT after IVT in patients with AIS. The integration of PWR into a nomogram model provides a practical tool for stratifying HT risk, potentially guiding individualized treatment strategies.

## 1 Introduction

In China, acute ischemic stroke (AIS) constitutes approximately 70–80% of all stroke cases and is associated with high disability and mortality rates (1, 2). Early reperfusion therapy represents the cornerstone of AIS management, with intravenous thrombolysis (IVT) with recombinant tissue plasminogen activator (rt-PA) serving as one of the most effective interventions to improve patient outcomes. Accumulating evidence from large-scale randomized clinical trials (RCTs) indicates that 35.4–52.4% of AIS patients treated with IVT achieve favorable functional outcomes (assessed by modified Rankin Scale [mRS] scores 0–1), a proportion significantly higher than that observed in control groups (3–5). However, hemorrhagic transformation (HT) following IVT has been documented in 27–37% of AIS cases (6), emerging as a critical determinant of post-thrombolysis prognosis (7).

The role of inflammation in thrombosis and cerebral ischemia is well-established, and it is also increasingly implicated in the pathophysiology of HT (8). Emerging evidence suggests that reperfusion-induced inflammation following IVT contributes to the occurrence of HT (9). Platelets (PLTs) and white blood cells (WBCs), key mediators of systemic inflammation, are involved in these processes, exacerbating vascular and cerebral tissue injury (10). Notably, the PLT-to-WBC ratio (PWR)—a composite peripheral hematologic index that reflects the host’s inflammatory response—has demonstrated superior prognostic value compared to other indices derived from complete blood counts in acute inflammatory conditions, including stroke (11). A lower PWR, indicative of leukocytosis and/or thrombocytopenia, has been significantly associated with short-term mortality in several cohorts (11).

Despite these advances, the relationship between PWR and HT, particularly its association with HT severity, remains insufficiently explored. Therefore, this study aims to investigate whether reduced PWR levels are correlated with an increased risk of HT and greater severity of HT. Besides, we utilized multivariable logistic regression to identify significant variables and developed a nomogram-based prediction model for HT risk stratification.

## 2 Method

### 2.1 Participants

We conducted a retrospective cohort study including AIS patients treated with IVT at Affiliated Jinhua Hospital, Zhejiang University School of Medicine between January 2018 and October 2024. The inclusion criteria were: (1) age of 18 years or older; (2) AIS diagnosis confirmed through computed tomography (CT) or magnetic resonance imaging (MRI) upon admission; and (3) administration of rt-PA at the standard dosage (0.9 mg/kg, with a maximum of 90 mg) within 4.5 h of symptom onset. The exclusion criteria were: (1) subsequent administration of endovascular therapy; (2) missing a follow-up brain scan (CT/MRI) within 24 h after IVT; (3) history of hematologic disorders or use of immunosuppressive medications; and (4) presence of an active infection within 2 weeks before hospitalization.

The study received approval from the Ethics Committee of Affiliated Jinhua Hospital, Zhejiang University, School of Medicine. The Ethics Committee of our hospital approved data collection from our stroke database and specifically granted a waiver of informed consent for this study due to the retrospective design and complete anonymity of patient records. All procedures in this study adhered to the Helsinki Declaration.

### 2.2 Data collection

We collected and documented the demographic data (age and gender); lifestyle risk factors for AIS (history of smoking and drinking); vascular risk factors for AIS: hypertension, diabetes mellitus (DM), atrial fibrillation (AF), coronary heart disease (CHD); clinical information: body mass index (BMI), systolic blood pressure (SBP) at admission, diastolic blood pressure (DBP) at admission, National Institutes of Health Stroke Scale (NIHSS) at admission, modified Rankin Scale (mRS) at discharge (12), onset to needle time (ONT), door to needle time (DNT), the time window, prior antithrombotic (antiplatelet and anticoagulant) drugs usage; laboratory indicators: PLT, WBC, hemoglobin, albumin, fasting glucose, low density lipoprotein (LDL) and high density lipoprotein (HDL). Blood samples were collected on the first morning after admission (at least 8 h for fasting). The severity of AIS was assessed by the NIHSS score at admission (13). All patients were classified based on the Trial of Org 10,172 in Acute Stroke Treatment (TOAST) criteria into the following etiologic subtypes: large-artery atherosclerosis, cardioembolism, small-vessel occlusion, and other subtypes (encompassing stroke of other determined etiology and undetermined etiology) (14). Peripheral venous blood samples were obtained after an overnight fast (≥8 h) on the morning following admission.

### 2.3 Assessment of PWR and HT

The PWR was calculated for each patient using the formula: PWR = PLT (10^9^/L)/WBC (10^9^/L). All patients with suspected AIS underwent a baseline cranial computed tomography CT scan before receiving IVT. Within 24 h following the IVT treatment, a cranial scan (CT/MRI) was performed to screen for HT. Besides, an immediate imaging examination was conducted whenever clinical symptom deterioration was observed. The diagnosis and classification of HT were based on the follow-up CT or MRI images. The radiological classification followed the established criteria of the European Cooperative Acute Stroke Study (ECASS) (15), categorizing HT into four distinct subtypes: hemorrhagic infarction (HI)-1 (small petechiae along the periphery of the infarct), HI-2 (more confluent petechiae around the infarcted area without a space-occupying effect), parenchymal hematoma (PH)-1 (hematoma <30% of the infarcted area with a mild space-occupying effect), and PH-2 (hematoma >30% of the infarcted area with a significant space-occupying effect).

### 2.4 Statistical analysis

Statistical analyses were conducted using R software, version 4.4.2. The Kolmogorov–Smirnov test was employed to assess the normality of the data distribution. Baseline characteristics were presented as mean ± standard deviation for normally distributed data, median with interquartile range for non-normally distributed data, and relative frequency with percentage for categorical variables. Continuous variables were analyzed using the Student’s *t*-test or Mann–Whitney *U* test, while categorical variables were assessed with the chi-square test or Fisher’s exact test. Patients in this study were categorized into four groups according to the quartiles of the PWR. Statistical comparisons of PWR stratification for continuous variables were conducted using either one-way ANOVA or the Kruskal–Wallis test, and Tukey’s HSD test was used as the post-hoc analysis for variables with significant differences. A multivariate logistic regression model was constructed to determine if PWR independently predicted HT. The model included covariates with *p* < 0.1 in univariate analysis and accounted for potential confounders. Besides, we applied restricted cubic spline (RCS) analysis to detect the nonlinear relationship of PWR and HT and PH risks. The correlations between PWR and ECASS subtypes were analyzed using Spearman’s rank correlation test. Nomograms were constructed using variables with *p*-values < 0.05 from multivariate logistic regression. Variance Inflation Factor (VIF) analysis was conducted to thoroughly assess potential multicollinearity among variables. A VIF threshold greater than 5 was employed to identify significant collinearity among the variables. The model’s regression coefficients were utilized to derive variable scores, forming the basis of the scoring system. The nomogram’s discriminative ability was evaluated using the area under the receiver operating characteristic (ROC) curve. The prediction model’s calibration, reflecting the concordance between observed and predicted probabilities via nomograms, was evaluated using 1,000 resampled calibration plots. A two-sided *p*-value < 0.05 was considered significant.

## 3 Results

### 3.1 Baseline characteristics

**Figure 1:**
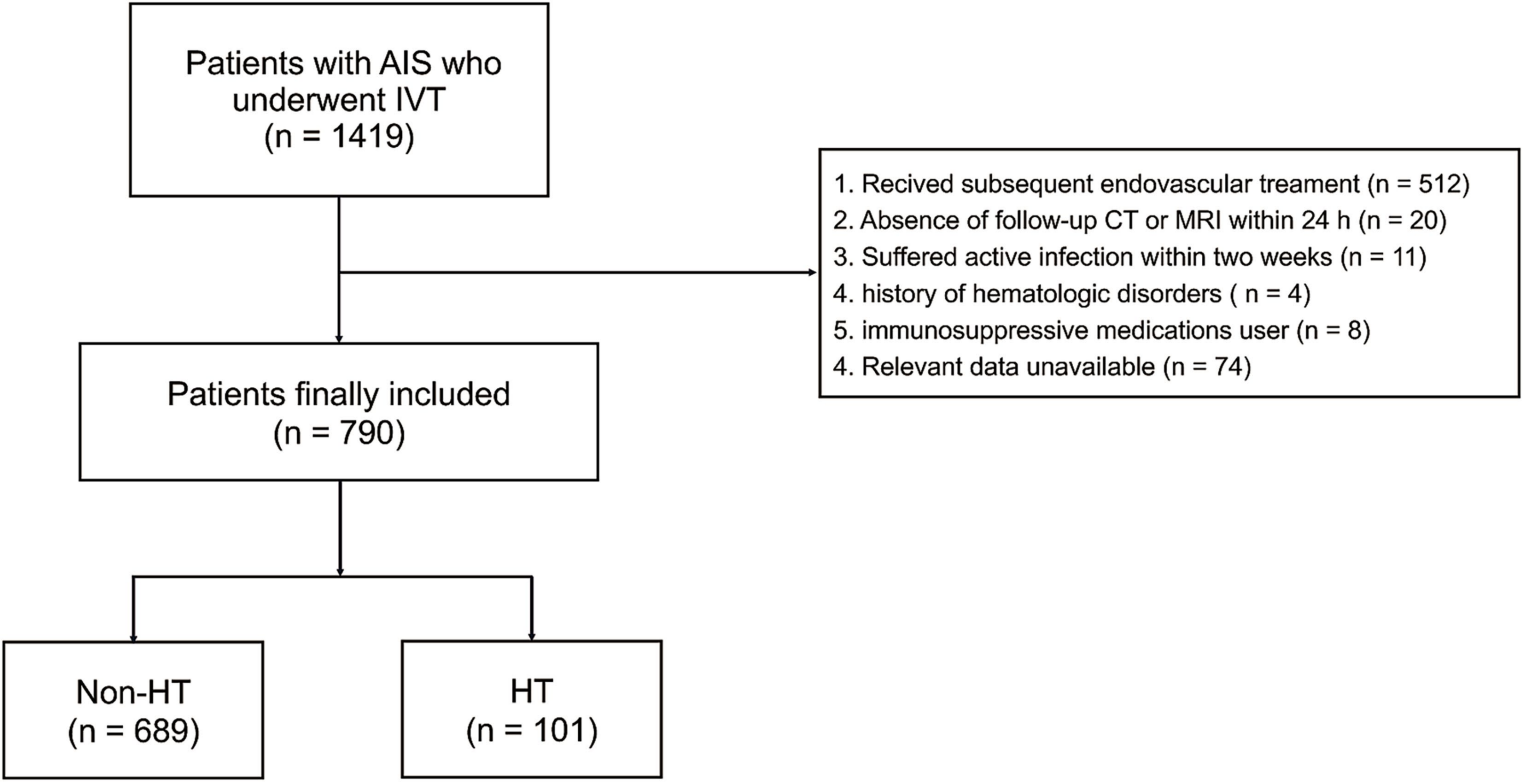
The flow chart of this study.

A total of 790 eligible AIS patients were finally included and analyzed (Figure 1). The study cohort comprised 514 males (65.0%) and had a median age of 70 years, ranging from 60 to 78 years. Among these, HT was developed in 101 patients (12.8%), whereas 690 patients (87.2%) did not. Key baseline and clinical characteristics included the NIHSS score at admission, with a median of 3.0 (1.0–6.0); the mRS score at discharge, with a median of 2.0 (1.0–3.0); 20.4% of all patients have concomitant with AF; ONT with a median of 105.0 (60.0–105.0) minutes; DNT, with a median of 38.0 (30.5–51.0) minutes; and the PWR, with a median of 28.2 (22.0–36.1). Patients were categorized into four groups according to PWR quartiles: Q1 (<22.0), Q2 (22.0–28.2), Q3 (28.3–36.2), and Q4 (>36.2). The incidence of HT was highest in the first quartile (Q1), and 48 (24.2%) patients developed HT. Notably, patients in the lower PWR quartiles were significantly older and presented with higher NIHSS scores upon admission, increased mRS scores at discharge, and elevated hemoglobin levels. Besides, these groups showed a higher prevalence of AF and reduced LDL levels (all *p* < 0.05). Detailed comparisons across quartiles are presented in Table 1.

**Table 1:** Comparison of baseline characteristics between patients according to PWR quartiles.

Variables	PWR quartiles					
	All patients (*n* = 790)	Quartile 1 (*n* = 198)	Quartile 2 (*n* = 198)	Quartile 3 (*n* = 197)	Quartile 4 (*n* = 197)	*p*-value
HT	101 (12.7%)	48 (24.2%)	18 (10.6%)	23 (10.1%)	12 (6.1%)	**<0.001**
Demographics						
Age, years (IQR)	70.0 (60.0–78.0)	74.0 (65.0–82.0)	70.0 (60.0–78.1)	69.0 (57.0–77.0)	67.0 (57.0–75.0)	**<0.001**
Gender (male, %)	514 (65.0%)	143 (72.2%)	143 (72.2%)	122 (61.9%)	106 (53.8%)	**<0.001**
Vascular risk factor						
Hypertension, *n* (%)	630 (79.7%)	154 (77.8%)	167 (84.3%)	155 (78.7%)	154 (78.2%)	0.320
DM, *n* (%)	173 (21.8%)	46 (23.2%)	46 (23.2%)	53 (26.9%)	44 (22.3%)	0.720
AF, *n* (%)	160 (20.2%)	67 (33.8%)	40 (20.2%)	34 (17.3%)	19 (9.6%)	**<0.001**
CHD, *n* (%)	56 (7.0%)	18 (9.1%)	17 (8.6%)	11 (5.6%)	10 (5.1%)	0.288
History of stroke, *n* (%)	119 (15.0%)	37 (18.7%)	33 (16.7%)	23 (11.7%)	26 (13.2%)	0.192
History of smoking, *n* (%)	324 (41.0%)	95 (48.0%)	86 (43.4%)	79 (40.1%)	64 (32.5%)	**0.015**
History of drinking, *n* (%)	344 (43.5%)	91 (46.0%)	92 (46.5%)	84 (42.3%)	77 (39.1%)	0.421
Clinical Information						
SBP, mmHg (IQR)	151.0 (136.0–167.0)	153.0 (139.0–171.0)	151.0 (135.3–167.0)	150.0 (136.0–164.0)	151.0 (135.0–165.0)	0.280
DBP, mmHg (IQR)	86.0 (76.0–95.8)	87.0 (75.3–96.0)	86.0 (76.0–96.0)	85.0 (75.0–95.0)	86.0 (77.0–94.0)	0.917
BMI, cm/kg^2^ (IQR)	23.3 (20.8–25.6)	22.8 (20.1–25.6)	23.9 (21.6–26.0)	23.3 (20.7–25.3)	22.8 (20.4–25.1)	**0.029**
NIHSS at admission (IQR)	3.0 (1.0–6.0)	4.5 (2.0–9.0)	3.0 (1.0–5.0)	2.0 (1.0–5.0)	2.0 (1.0–4.0)	**<0.001**
mRS at discharge (IQR)	2.0 (1.0–3.0)	3.0 (1.0–4.0)	2.0 (1.0–3.0)	2.0 (1.0–3.0)	1.0 (1.0–2.0)	**<0.001**
Time window						**0.017**
<3.0 h, *n* (%)	640 (81.0%)	162 (81.8%)	169 (85.4%)	164 (83.2%)	145 (73.6%)	
3.0–4.5 h, *n* (%)	150 (18.9%)	36 (18.2%)	29 (14.6%)	33 (16.8%)	52 (26.4%)	
ONT, min (IQR)	105.0 (60.0–105.0)	98.0 (60.0–155.3)	101.0 (60.0–141.3)	100.5 (65.0–147.0)	120.0 (64.0–180.0)	**0.048**
DNT, min (IQR)	38.0 (30.0–51.0)	38.0 (30.0–50.0)	38.0 (30.0–50.8)	38.0 (30.0–51.0)	40 (29.0–55.0)	0.918
Prior antiplatelet/anticoagulant use	172 (21.7%)	56 (28.3%)	41 (20.7%)	31 (15.7%)	44 (22.3%)	**0.025**
TOAST classification						**<0.001**
Large artery atherosclerosis, *n* (%)	258 (32.6%)	61 (30.8%)	69 (34.8%)	65 (33.0%)	63 (32.0%)	
Cardioembolism, *n* (%)	173 (21.8%)	73 (36.9%)	44 (22.2%)	35 (17.8%)	21 (10.7%)	
Small vessel occlusion, *n* (%)	279 (35.3%)	45 (22.7%)	61 (30.8%)	78 (39.6%)	95 (48.2%)	
Others, *n* (%)	80 (10.1%)	19 (9.6%)	24 (12.1%)	19 (9.6%)	18 (9.1%)	
Laboratory indictors						
Platelet, 10^9^/L (IQR)	199.0 (154.3–239.8)	145.5 (124.0–184.0)	191.5 (156.3–224.8)	208.0 (175.0–246.0)	241.0 (208.0–286.0)	**<0.001**
WBC, 10^9^/L (IQR)	7.0 (5.7–8.6)	8.8 (7.2–10.7)	7.7 (6.0–8.9)	6.6 (5.6–7.6)	5.6 (4.8–6.7)	**<0.001**
PWR, (IQR)	28.2 (22.0–36.1)	18.3 (15.4–20.2)	25.4 (23.8–26.9)	31.5 (29.8–33.8)	41.3 (38.5–46.3)	**<0.001**
Hemoglobin, g/L (IQR)	135.5 (124.0–147.0)	136.0 (121.0–148.8)	138.0 (128.3–149.0)	135.0 (125.0–145.0)	133.0 (121.0–144.0)	**0.015**
Albumin, g/L (IQR)	38.0 (35.9–40.2)	37.5 (35.3–39.3)	37.9 (35.9–40.4)	38.6 (36.3–39.9)	38.3 (36.2–40.5)	**0.035**
Fasting glucose, mmol/L (IQR)	5.1 (4.6–6.1)	5.5 (4.6–6.5)	5.1 (4.6–6.1)	5.2 (4.6–6.2)	5.1 (4.6–5.7)	0.223
LDL, mmol/L (IQR)	2.8 (2.3–3.4)	2.6 (2.1–3.1)	2.8 (2.3–3.4)	3.0 (2.4–3.5)	3.0 (2.5–3.5)	**<0.001**
HDL, mmol/L (IQR)	1.2 (2.3–3.4)	1.1 (1.0–1.4)	1.1 (1.0–1.4)	1.2 (1.0–1.4)	1.2 (1.0–1.4)	0.393

HT, hemorrhagic transformation; DM, diabetes mellitus; AF, atrial fibrillation; CHD, coronary heart disease; SBP, systolic blood pressure; DBP, diastolic blood pressure; BMI, body mass index; NIHSS, National Institutes of Health Stroke Scale; mRS, modified Rankin Scale; ONT, onset to needle time; WBC, white blood cell; PWR, platelet to white blood cell ratio; LDL, low density lipoprotein; HDL, high density lipoprotein. *p*-value < 0.05 was marked as bold.

### 3.2 The association between PWR and HT

**Figure 2:**
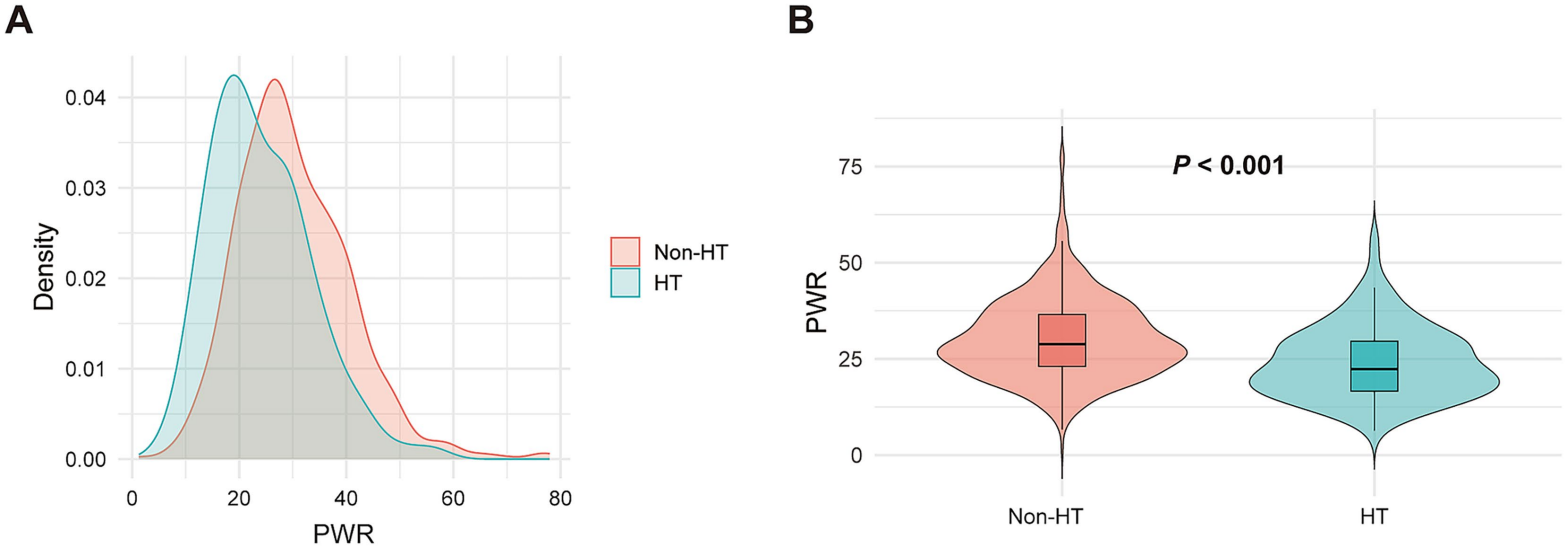
Probability density curve and violin plot of the PWR showing the differences between HT group and Non-HT group. **(A)** Probability density plot of PWR; **(B)** Violin plot of PWR.

When PWR was analyzed as a continuous variable, patients without HT had higher PWR levels than patients with HT (22.1 [16.6–29.0] vs. 29.0 [23.2–36.5], *p* < 0.001; Figure 2). When treated with a categorical variable, the percentage of HT progressively declined from Q1–Q4 (dropped from 24.2% in Q1 to 6.1% in Q4, *p* < 0.001). Table 2 presents the logistic regression results of the association between PWR and HT in AIS patients after IVT. In the univariate analysis, patients in groups Q2, Q3, and Q4 had a reduced HT risk compared with those in Q1. In the multivariable model, after adjusting for confounding variables (*p* < 0.1 in univariable analysis, Supplementary Table 1), Q2, Q3 and Q4 remained significantly associated with a decreased risk of HT (Q2: odds ratio [OR]: 0.506, 95% confidence interval [CI]: 0.303–0.833, *p* = 0.015; Q3: OR: 0.526, 95% CI: 0.310–0.879, *p* = 0.042 and Q3: OR: 0.333, 95% CI: 0.176–0.606, *p* = 0.003; Table 2).

**Table 2:** Multivariate logistic regression of the association between PWR and HT.

Variables	Crude model		Model 2		Model 3	
	OR (95%CI)	*P*-value	Adjusted OR (95%CI)	*P*-value	Adjusted OR (95%CI)	*P*-value
As continuous	0.928 (0.908–0.948)	**<0.001**	0.937 (0.917–0.958)	**<0.001**	0.950 (0.928–0.971)	**<0.001**
Q1: <22.0	Ref		Ref		Ref	
Q2: 22.0–28.3	0.371 (0.230–0.586)	**<0.001**	0.426 (0.260–0.687)	**0.003**	0.506 (0.303–0.833)	**0.026**
Q3: 28.3–36.2	0.353 (0.217–0.562)	**<0.001**	0.426 (0.256–0.696)	**0.005**	0.526 (0.310–0.879)	**0.042**
Q4: >36.2	0.203 (0.112–0.347)	**<0.001**	0.250 (0.135–0.444)	**<0.001**	0.333 (0.176–0.606)	**0.003**

PWR, platelet to white blood cell ratio; HT, hemorrhagic transformation.

Crude model did not adjust any confounders.

Model 2 adjusted for age and gender, atrial fibrillation, history of smoking, history of drinking.

Model 3 adjusted for covariates from Model 2 and further adjusted for NIHSS at admission, hemoglobin, LDL and HDL.

*p*-value < 0.05 was marked as bold.

**Figure 3:**
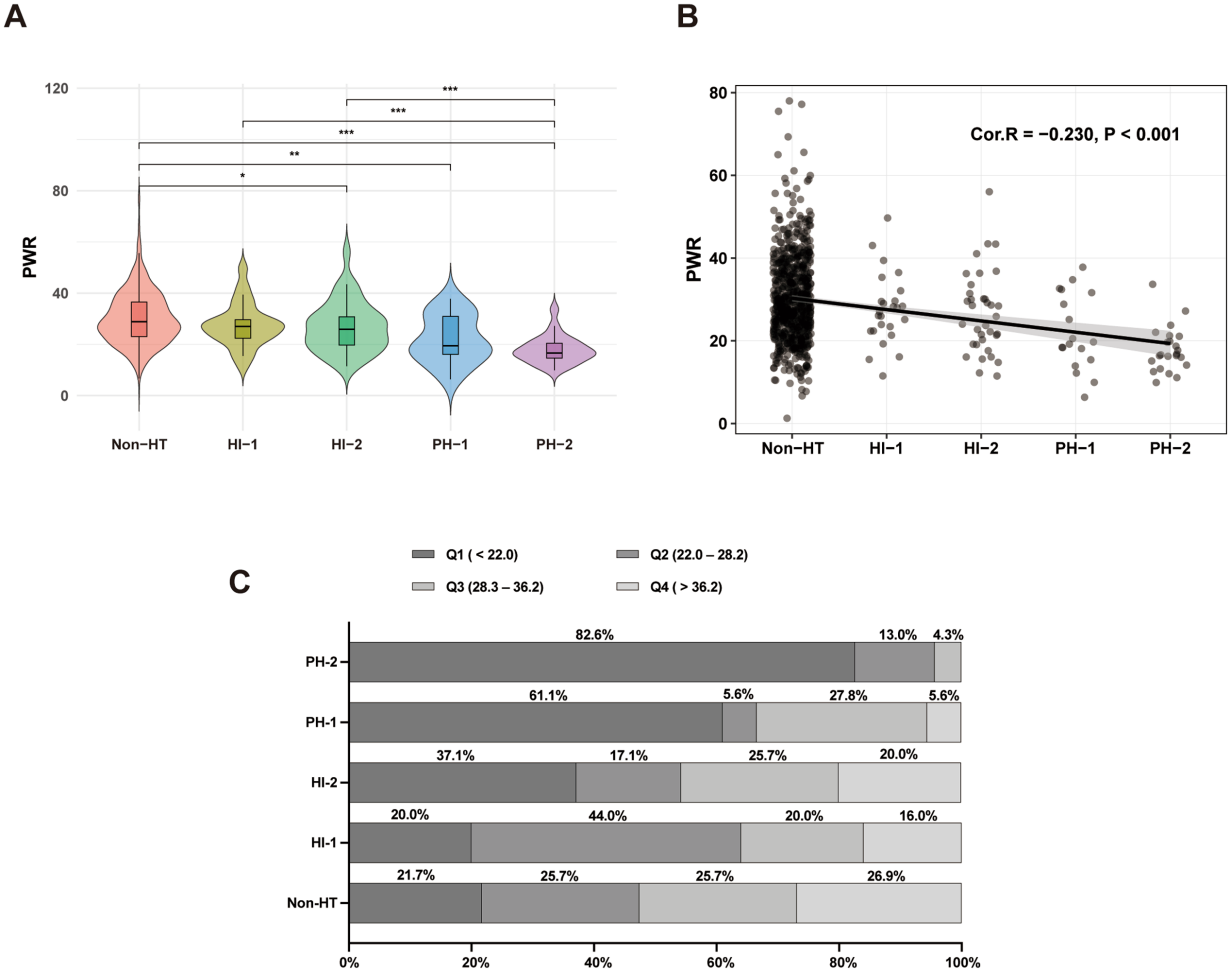
The relationship between PWR and HT subtypes. **(A)** PWR in different HT subtypes. **(B)** The negative relationship between PWR and HT subtypes. **(C)** Proportion of patients in each PWR with different HT subtypes.

**Figure 4:**
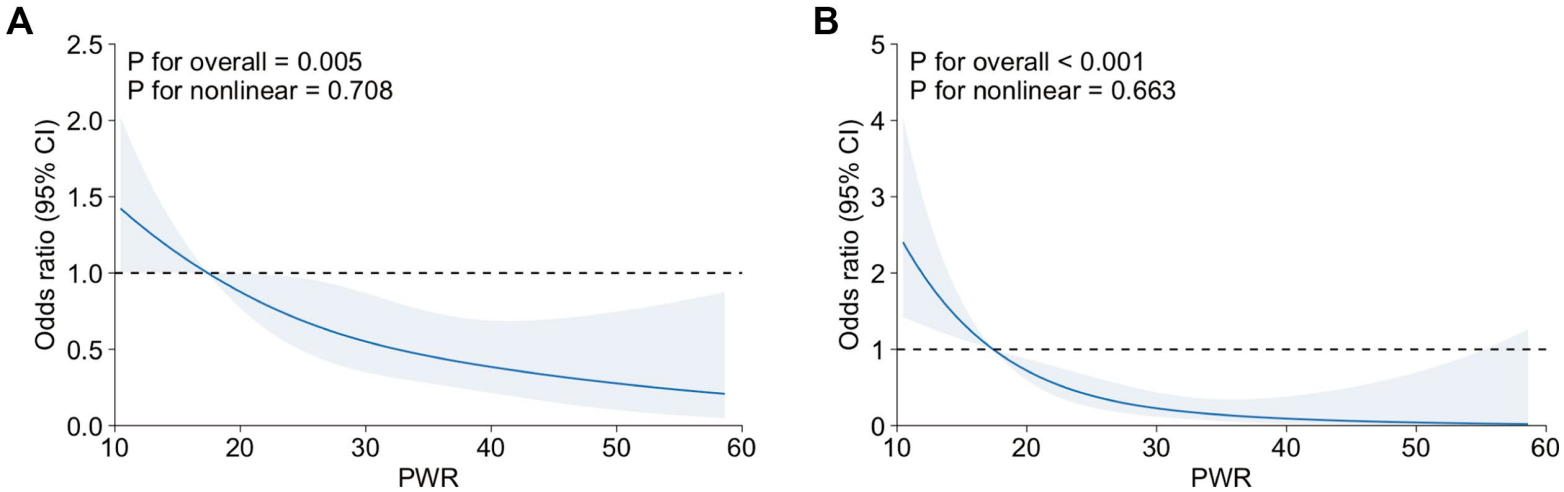
The multivariable restricted cubic spline (RCS) analysis for PWR in **(A)** HT and **(B)** PH. The solid blue line represents the odds ratio (OR), and the blue shading depicts the corresponding 95% confidence interval (CI). The dotted black line is the reference line for which OR = 1. The model was adjusted for the same covariates in Model 3.

The examination of PWR quartiles in relation to HT subtypes indicated that all subtypes, except HI-1, had decreased PWR levels compared to non-HT, with significant differences between PH-2 and HI-1, and PH-2 and HI-2 (*p* < 0.001), as shown in Figure 3A. A negative association was found between elevated PWR score and HT severity (Spearman correlation coefficient: −0.230, *p* < 0.001) (Figure 3B). Furthermore, the proportion of patients with the lowest PWR quartile (Q1) increased markedly with HT severity, accounting for 82.6% of PH-2 cases compared to only 20.0% of HI-1 cases (Figure 3C). The RCS analysis revealed that the association between PWR and HT or PH was confirmed to be a linear relationship (*p* > 0.1 for non-linearity, Figure 4).

### 3.3 The construction of the nomogram

**Figure 5:**
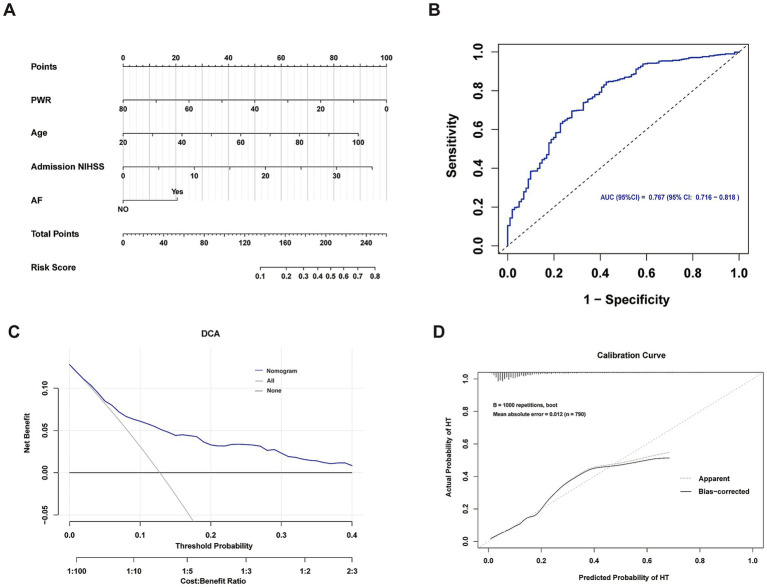
Development and validation of the Nomogram for predicting HT. **(A)** Nomogram. **(B)** Receiver operating characteristic (ROC) curve assessing the discriminatory ability of the Nomogram. **(C)** Decision curve analysis (DCA) evaluating the clinical utility of the model. **(D)** Calibration curve comparing predicted probabilities and observed HT.

The nomogram was constructed by incorporating the independent predictors identified in Table 3, resulting in a graphical tool for personalized risk assessment, as illustrated in Figure 5A. The prediction model is as follows: logit(*P*) = −3.265 + (0.027 × Age) + (0.094 × NIHSS) + (0.614 × AF) + (−0.338 × *P*WR). The predicted probability of HT is then calculated as:


$$P=\frac{e^{\text{logit}(P)}}{1+e^{\text{logit}(P)}}$$

**Table 3:** The multivariable analyses of risk factors for predicting HT.

Variables	OR (95% CI)	*P*-value	VIF
PWR	0.953 (0.932–0.974)	**<0.001**	1.085
Age	1.036 (1.019–1.054)	**<0.001**	1.086
Admission NIHSS	1.091 (1.057–1.126)	**<0.001**	1.063
Atrial fibrillation	1.906 (1.253–2.878)	**0.011**	1.103

HT, hemorrhagic transformation; NIHSS, National Institutes of Health Stroke Scale; VIF, variance inflation factor. *p*-value < 0.05 was marked as bold.

The VIF analysis showed that all predictors were considered presence of weak collinearity among these variables, thereby affirming their appropriateness for further analysis (VIF < 5 for all variables).

To utilize the nomogram, one draws a vertical line from each predictor value to the ‘Total Points’ scale to obtain an individual score. The aggregate of these scores produces a total point value, which is then aligned with the probability scale to ascertain the corresponding probability of HT. The model’s predictive performance was assessed using ROC with an AUC of 0.767 (95% CI: 0.716–0.818) (Figure 5B). Additionally, DCA demonstrated that the nomogram provides a net benefit over various threshold probabilities, underscoring its potential for clinical decision-making (Figure 5C). The calibration curve showed strong agreement between predicted HT probabilities and actual outcomes (mean absolute error = 0.012), indicating the model’s effective calibration for estimating HT likelihood following IVT (Figure 5D).

## 4 Discussion

This study analyzed data from a tertiary hospital to determine the effect of PWR on HT risk in AIS patients treated with IVT. The study found a significant association between lower PWR levels and an increased risk of HT. Furthermore, a lower PWR level was linked to more severe HT subtypes compared to a higher level. Besides, we developed a nomogram that exhibited potential discrimination and calibration. According to the DCA results, the predictive model provided a substantial net benefit in assessing HT risk in AIS patients following IVT.

This study indicates that 12.7% of participants developed HT, consistent with the 10–43% range documented in earlier research (16). Our results are consistent with research on HT risk factors after IVT conducted previously, which found that age, NIHSS score at admission, and AF might impact the incidence of HT after IVT. Besides, this study shows that PWR is one of the most common risk factors found for HT in AIS patients post-IVT.

Nomogram is widely used in clinical diagnosis and prognosis prediction. This study utilized logistic regression to integrate age, NIHSS score at admission, AF, and PWR into a nomogram, achieving a notable predictive performance with an AUC of 0.767. Age is widely recognized as an independent risk factor for HT following IVT and as a prognostic factor for AIS, as confirmed by numerous studies (17, 18). Dong et al.’s (19) meta-analysis of 25 cohort studies identified advanced age as an independent predictor of HT in AIS patients. The NIHSS score at admission independently affects both short-term and long-term prognosis in AIS patients (20, 21). Therefore, a substantial number of nomogram models have included the NIHSS score as an important variable for HT prediction (22, 23). AF, a prevalent arrhythmia in clinical practice, increases the risk of HT in AIS patients (24). The mechanisms underlying this increased risk are multifactorial. First, cardiogenic thrombi in AF patients are often older and more organized, which may reduce their sensitivity to IVT, leading to prolonged time to recanalization and consequently increasing the risk of HT (25). Second, AF-related AIS often presents with poor collateral circulation, severe intracranial hypoperfusion, and larger infarct volumes, which increase the neurovascular unit’s susceptibility to reperfusion injury and hemorrhage (24). Furthermore, a history of anticoagulant use in a subgroup of AF patients prior to IVT represents an additional, significant risk factor for HT.

Several studies have reported links between PWR and prognosis in different cerebrovascular diseases, which is consistent with our research findings (26, 27). Elevated PWR is significantly positively associated with prognosis in patients with AIS, intracerebral hemorrhage (ICH), and subarachnoid hemorrhage (SAH). Patients with aneurysmal SAH and low preoperative PWR are at a higher risk of postoperative pneumonia (27). However, the association between the PWR and the HT after IVT has not yet been fully established and needs to be elucidated. HT involves more than just the mechanical rupture of cerebral vessels; it is a complex process characterized by inflammatory-mediated blood–brain barrier (BBB) disruption (28), matrix degradation (29), and altered coagulation status (30). WBCs are essential for inflammation resolution and tissue repair. Elevated WBC levels are consistently linked to larger infarction volumes, faster neurological decline, and poor functional outcomes in AIS patients (31). In the context of stroke progression, PLTs are involved in both hemostasis and the preservation of the BBB, yet they also have proinflammatory effects that can negatively impact stroke outcomes (32). Inflammation developed from AIS worsens initial brain damage and hinders later recovery (33). Research indicates that PLT depletion due to cerebral ischemia–reperfusion also worsens brain tissue damage (34). The acute inflammatory response is closely associated with HT after IVT. This association stems from a thrombo-inflammatory process initiated during the acute phase: PLTs guide lymphocytes to sites of vascular injury, while T cells (a lymphocyte subgroup) release cytokines that regulate PLT activation. This cascade can compromise tissue integrity, disrupt the BBB, and increase oxidative stress (35). Therefore, the acute inflammatory response is closely associated with post-IVT HT.

The pathophysiological link between PWR and HT risk after IVT is plausibly rooted in the dynamic interplay between inflammation and hemostasis processes that PWR uniquely encapsulates. Recently, Foy et al. (11) found that the recovery trajectory from an acute inflammatory insult is characterized by a coordinated decrease in WBC, signifying resolution of the initial inflammatory surge, followed by a linear increase in PLT, which indicates successful hematopoietic recovery. However, a low PWR, therefore, signifies a deviation from this healthy process, representing either a persistent, dysregulated inflammatory state (elevated WBC), a failure of PLT reconstitution (decreased PLT), or both. While both PLTs and WBCs are individually implicated in post-IVT HT, the PWR offers a distinct physiological advantage by capturing the dynamic balance between hemostasis and inflammation. In the context of AIS, a low PWR at admission may reflect an exaggerated and poorly controlled inflammatory response to cerebral ischemia. This heightened inflammation can exacerbate BBB permeability and release proteolytic enzymes, creating a microenvironment conducive to vascular leakage and HT upon reperfusion (36, 37).

Nonetheless, this research has certain limitations. First, as a single-center, retrospective cohort analysis, the findings demonstrate an association but cannot establish causality, and the results may be influenced by inherent selection biases and unmeasured confounding factors. Second, PWR was collected only once at hospital admission. This single measurement does not account for potential fluctuations in PWR before and after IVT or during the hospitalization period, which might provide a more dynamic and accurate risk assessment. Lastly, the sample size of HT in this study was relatively small. To address these limitations, further prospective multicenter studies are necessary to confirm the link between PWR and HT following IVT.

## 5 Conclusion

In conclusion, this study suggests that a lower PWR is associated with a higher occurrence of HT and more severe subtypes after IVT in AIS patients. By integrating potential risk factors, including PWR, the nomogram achieved a good discriminative ability. These findings may help clinicians identify high-risk individuals with HT after IVT and provide some assistance to clinicians in clinical decision-making after thrombolysis in AIS patients.

## Data Availability

The original contributions presented in the study are included in the article/Supplementary material, further inquiries can be directed to the corresponding author.
