# Supplementary material for: Nomogram-based prediction of hemorrhagic transformation risk integrating platelet-to-white blood cell ratio in patients with acute ischemic stroke after intravenous thrombolysis
Source: Front Neurol. 2026 Jun 17;17:1782110. doi: 10.3389/fneur.2026.1782110 (PMC13318647; doi:10.3389/fneur.2026.1782110)
Supplement: Supplementary file 1 [file Table_1.docx]

Supplementary table 1: Univariate logistic regression analysis for covariates.

| Variables | OR (95% CI) | P value |
| --- | --- | --- |
| Age | 1.055 (1.038-1.073) | **<0.001** |
| Gender (female) | 1.593 (1.115-2.270) | **0.031** |
| Hypertension | 1.294 (0.828-2.101) | 0.361 |
| Diabetes mellitus | 0.870 (0.562-1.313) | 0.589 |
| Atrial ﬁbrillation | 3.445 (2.370-4.988) | **<0.001** |
| CHD | 0.807 (0.361-1.595) | 0.631 |
| History of stroke | 0.733 (0.416-1.220) | 0.340 |
| History of smoking | 0.599 (0.407-0.867) | **0.025** |
| History of drinking | 0.652 (0.449-0.936) | **0.055** |
| Time window | 1.393 (0.907-2.094) | 0.192 |
| SBP | 1.005 (0.997-1.013) | 0.334 |
| DBP | 0.998 (0.986-1.009) | 0.748 |
| Body mass index | 0.954 (0.907-1.002) | 0.118 |
| NIHSS at admission | 1.128 (1.095-1.163) | **<0.001** |
| DNT | 1.000 (0.993-1.007) | 0.946 |
| OTT | 1.001 (0.999-1.003) | 0.378 |
| TOAST | 0.845 (0.708-1.005) | 0.113 |
| Prior antiplatelet/anticoagulant use | 0.892 (0.563-1.362) | 0.663 |
| Hemoglobin | 0.983 (0.974-0.993) | **0.005** |
| Albumin | 0.985 (0.940-1.031) | 0.581 |
| Fasting glucose | 0.992 (0.951-1.002) | 0.483 |
| PLT | 0.994 (0.991-0.997) | **0.001** |
| WBC | 1.210 (1.131-1.294) | **<0.001** |
| PWR | 0.928 (0.908-0.948) | **<0.001** |
| LDL | 0.670 (0.531-0.839) | **0.004** |
| HDL | 1.528 (1.023-2.245) | **0.069** |

**Abbreviations**: OR, odds ratio; CI, confidence interval; CHD, coronary heart disease; SBP, systolic blood pressure; DBP, diastolic blood pressure; NIHSS, national Institutes of Health Stroke Scale; DNT, door to needle time; ONT, onset to needle time; TOAST, Trial of ORG 10172 in Acute Stroke Treatment; PLT, platelet; WBC, white blood cell; PWR, platelet to white blood cell ratio; LDL-C, low-density lipoprotein cholesterol; HDL-C, high-density lipoprotein cholesterol

**Note**: variables with P < 0.1 were marked as bold and included in subsequent multivariable analysis
